# Epistasis Test in Meta-Analysis: A Multi-Parameter Markov Chain Monte Carlo Model for Consistency of Evidence

**DOI:** 10.1371/journal.pone.0152891

**Published:** 2016-04-05

**Authors:** Chin Lin, Chi-Ming Chu, Sui-Lung Su

**Affiliations:** 1 Graduate Institute of Life Sciences, National Defense Medical Center, Taipei, Taiwan, ROC; 2 School of Public Health, National Defense Medical Center, Taipei, Taiwan, ROC; Inc, UNITED STATES

## Abstract

Conventional genome-wide association studies (GWAS) have been proven to be a successful strategy for identifying genetic variants associated with complex human traits. However, there is still a large heritability gap between GWAS and transitional family studies. The “missing heritability” has been suggested to be due to lack of studies focused on epistasis, also called gene–gene interactions, because individual trials have often had insufficient sample size. Meta-analysis is a common method for increasing statistical power. However, sufficient detailed information is difficult to obtain. A previous study employed a meta-regression-based method to detect epistasis, but it faced the challenge of inconsistent estimates. Here, we describe a Markov chain Monte Carlo-based method, called “Epistasis Test in Meta-Analysis” (ETMA), which uses genotype summary data to obtain consistent estimates of epistasis effects in meta-analysis. We defined a series of conditions to generate simulation data and tested the power and type I error rates in ETMA, individual data analysis and conventional meta-regression-based method. ETMA not only successfully facilitated consistency of evidence but also yielded acceptable type I error and higher power than conventional meta-regression. We applied ETMA to three real meta-analysis data sets. We found significant gene–gene interactions in the renin–angiotensin system and the polycyclic aromatic hydrocarbon metabolism pathway, with strong supporting evidence. In addition, glutathione *S*-transferase (GST) mu 1 and theta 1 were confirmed to exert independent effects on cancer. We concluded that the application of ETMA to real meta-analysis data was successful. Finally, we developed an R package, etma, for the detection of epistasis in meta-analysis [etma is available via the Comprehensive R Archive Network (CRAN) at https://cran.r-project.org/web/packages/etma/index.html].

## Introduction

Many complex human traits are considered to be associated with genetic factors, and previous genetic studies have identified a large number of causal variants [1]. However, the sum of the estimated genetic effects has often been much less than the heritability of the trait, a phenomenon called ‘missing heritability’ [2]. This ‘missing heritability’ is often attributed to the technical limitations of epistasis estimation [3–5]. Generally, the most important limitation is sample size. A single study is often ineffective for detecting epistasis [3,6].

Meta-analysis has become a popular method for discovering genetic risk variants, because it can increase detection power [7,8]. However, few studies have sought to detect epistasis [9], because sufficient detailed information is difficult to obtain [10]. The frequencies of genotype combinations in case and control groups are needed for analysis of epistasis by current technology, but most published articles report only genotype frequencies. Thus, reported meta-analysis studies aiming at epistasis detection have been able to use only 20% of the reported studies [11–13]. The largest challenge of epistasis assessment in meta-analysis is the incompleteness of information.

**Fig 1:**
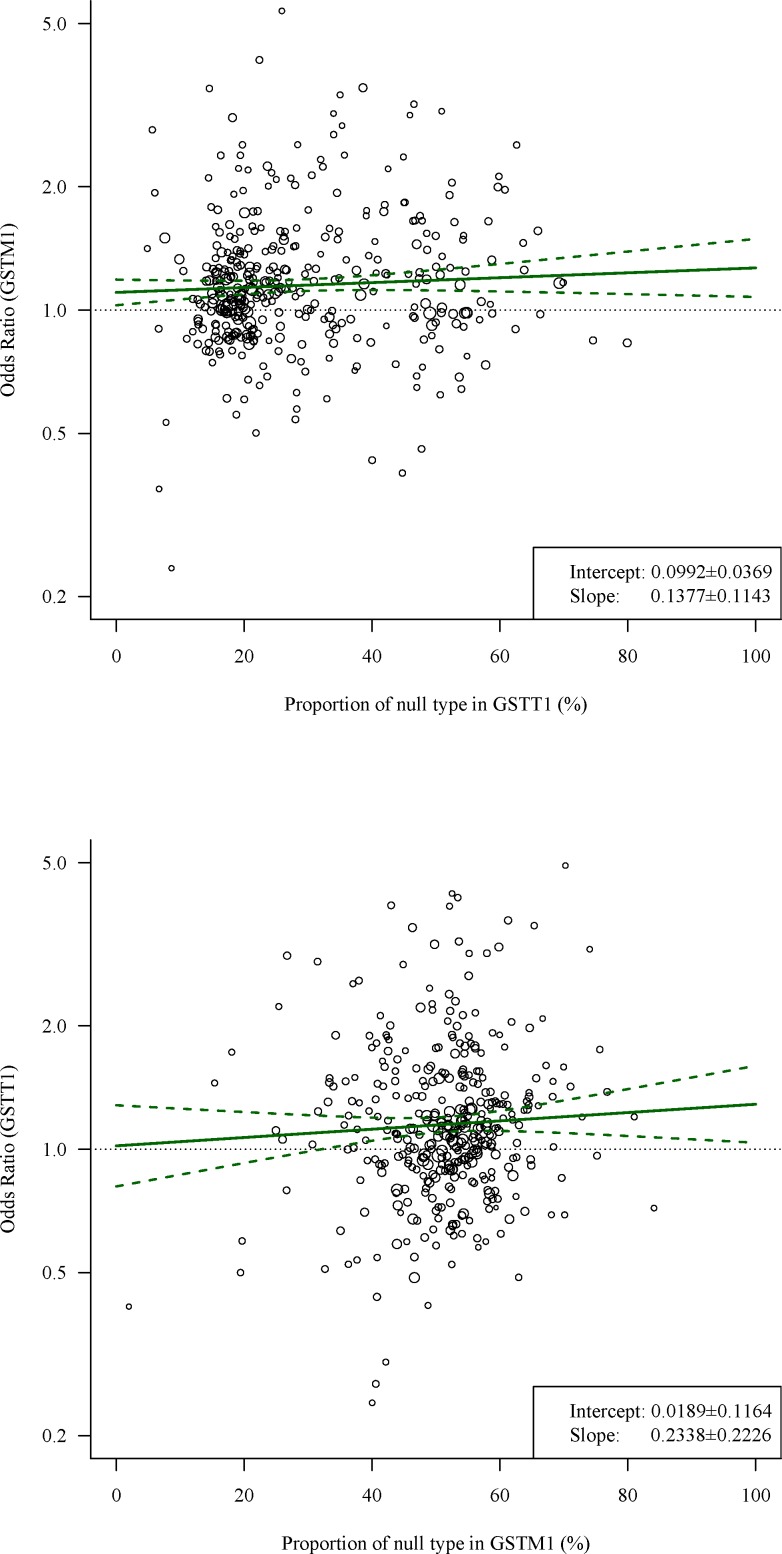
Inconsistent estimates of interaction effects in the same data. This figure describes a meta-regression analysis based on the data from Fang et al. [27] (detailed data are shown in S1 Table). The upper plot describes an investigation of the association between proportions of null/null GSTT1 in cases and the odds ratios of GSTM1 in cancer, and the lower plot describes an investigation of the association between proportions of null/null GSTM1 in cases and the odds ratios of GSTT1 in cancer. The solid lines denote unbiased estimators of odds ratios, and the dashed lines show 95% confidence intervals of odds ratios. According to a previous article, the slopes in meta regression approximate interaction effects [16]. However, the estimates of interaction effect were inconsistent when we exchanged the independent and moderator variables (0.1377 and 0.2338, respectively). This phenomenon does not occur in individual data analysis and leads to problems in interpretation.

Meta-regression is a common approach to assessing interaction effects in meta-analysis of randomised controlled trials [14,15], and a previous study popularised this method in meta-analysis of genetic association studies [16]. However, the inherent limitations of meta-regression have caused some problems in application of epistasis detection. The most important problem is attenuation bias. The average summary values in each included study are calculated from a small sample size and may thus include large random error [17–19]. Moreover, previous studies considered that two assumptions, rare disease and independence between SNPs, are necessary conditions for a linear relationship [16]. The rare-disease assumption is sometimes difficult to justify, and a previous study found slight error when this assumption was violated [16]. These random errors will lead to inconsistent estimates of interaction effects (see Fig 1), but this phenomenon does not occur in individual data analysis. Inconsistent evidence leads to difficulties in interpretation.

In summary, a single trial often has insufficient sample size, but meta-analysis lacks sufficient detailed individual information. The current method using averaged summary data for detecting interaction effects faces the challenge of inconsistent estimates. We propose a Markov chain Monte Carlo (MCMC)-based method, called ‘Epistasis Test in Meta-Analysis (ETMA)’, using genotype summary data for obtaining consistent estimates of epistasis in meta-analyses.

## Materials and Methods

### Derivations and description of ETMA

We assume that SNP1 (*x*_1_) and SNP2 (*x*_2_) are binary variables encoded as 0 and 1 (wild type and mutation, respectively), and that the dependent variable (*y*) is an outcome event encoded as 0 and 1 (health and disease, respectively). Under the above assumptions, we defined *p*_1_, *p*_2_, *p*_3_, *p*_4_, *p*_5_ and *p*_6_ as follows:

1. Disease risk in subjects with wild-type alleles of SNP1 and SNP2 (*p*_1_):
  $$p_{1}=p\left(y=1|x_{1}=0\cap x_{2}=0\right)$$
2. Disease risk in subjects with wild-type alleles of SNP1 and mutation of SNP2 (*p*_2_):
  $$p_{2}=p\left(y=1|x_{1}=0\cap x_{2}=1\right)$$
3. Disease risk in subjects with mutations of SNP1 and wild type of SNP2 (*p*_3_):
  $$p_{3}=p\left(y=1|x_{1}=1\cap x_{2}=0\right)$$
4. Disease risk in subjects with mutations of SNP1 and SNP2 (*p*_4_):
  $$p_{4}=p\left(y=1|x_{1}=1\cap x_{2}=1\right)$$
5. Mutation frequency of SNP1 (*p*_5_):
  $$p_{5}=p\left(x_{1}=1\right)$$
6. Mutation frequency of SNP2 (*p*_6_):
  $$p_{6}=p\left(x_{2}=1\right).$$

If *x*_1_ and *x*_2_ are independent, the above six parameters determine the distribution of *x*_1_, *x*_2_ and *y* in any population. However, we consider *p*_1_, *p*_5_ and *p*_6_ as population-specific parameters and define three constant parameters as follows:

1. Main effect of SNP1 on *y* (OR_y,SNP1_):
  $${\text{OR}}_{\;y,\text{SNP}1}=\frac{p_{3}\left(1-p_{1}\right)}{p_{1}\left(1-p_{3}\right)}$$
2. Main effect of SNP2 on *y* (OR_y,SNP2_):
  $${\text{OR}}_{\;y,\text{SNP}2}=\frac{p_{2}\left(1-p_{1}\right)}{p_{1}\left(1-p_{2}\right)}$$
3. Gene–gene interaction effect between SNP1 and SNP2 on *y* (OR_interaction_):
  $${\text{OR}}_{\;\text{interaction}}=\frac{p_{1}p_{4}\left(1-p_{2}\right)\left(1-p_{3}\right)}{p_{2}p_{3}\left(1-p_{1}\right)\left(1-p_{4}\right)}$$

Thus, *p*_2_, *p*_3_ and *p*_4_ can be calculated by the following equations:

$$p_{2}=\frac{p_{1}{\text{OR}}_{\;y,\text{SNP}2}}{p_{1}{\text{OR}}_{\;y,\text{SNP}2}+\left(1-p_{1}\right)}\;(2.1-1)$$

$$p_{3}=\frac{p_{1}{\text{OR}}_{\;y,\text{SNP}1}}{p_{1}{\text{OR}}_{\;y,\text{SNP}1}+\left(1-p_{1}\right)}\;(2.1-2)$$

$$p_{4}=\frac{p_{1}{\text{OR}}_{\;y,\text{SNP}1}{\text{OR}}_{\;y,\text{SNP}2}{\text{OR}}_{\;\text{interaction}}}{p_{1}{\text{OR}}_{\;y,\text{SNP}1}{\text{OR}}_{\;y,\text{SNP}2}{\text{OR}}_{\;\text{interaction}}+\left(1-p_{1}\right)}\;(2.1-3)$$

A case–control study including two loci often provides four exposure rates: (1) of the *x*_1_ mutation in the case group (*e*_case,x1_), (2) of the *x*_1_ mutation in the control group (*e*_ctrl,x1_), (3) of the *x*_2_ mutation in the case group (*e*_case,x2_) and (4) of the *x*_2_ mutation in the control group (*e*_ctrl,x2_). These four exposure rates can be represented as combinations of *p*_1_, *p*_2_, *p*_3_, *p*_4_, *p*_5_ and *p*_6_. Their relationships are shown as follows (detailed calculations are shown in S1 Text):

$$e_{\text{case},x1}=\frac{\left[p_{3}\left(1-p_{6}\right)+p_{4}p_{6}\right]p_{5}}{p_{1}\left(1-p_{6}\right)\left(1-p_{5}\right)+p_{2}p_{6}\left(1-p_{5}\right)+p_{3}\left(1-p_{6}\right)p_{5}+p_{4}p_{6}p_{5}}$$

$$e_{\text{ctrl},x1}=\frac{\left[\left(1-p_{3}\right)\left(1-p_{6}\right)+\left(1-p_{4}\right)p_{6}\right]p_{5}}{\left(1-p_{1}\right)\left(1-p_{6}\right)\left(1-p_{5}\right)+\left(1-p_{2}\right)p_{6}\left(1-p_{5}\right)+\left(1-p_{3}\right)\left(1-p_{6}\right)p_{5}+\left(1-p_{4}\right)p_{6}p_{5}}$$

$$e_{\text{case},x2}=\frac{\left[p_{2}\left(1-p_{5}\right)+p_{4}p_{5}\right]p_{6}}{p_{1}\left(1-p_{6}\right)\left(1-p_{5}\right)+p_{2}p_{6}\left(1-p_{5}\right)+p_{3}\left(1-p_{6}\right)p_{5}+p_{4}p_{6}p_{5}}$$

$$e_{\text{ctrl},x2}=\frac{\left[\left(1-p_{2}\right)\left(1-p_{5}\right)+\left(1-p_{4}\right)p_{5}\right]p_{6}}{\left(1-p_{1}\right)\left(1-p_{6}\right)\left(1-p_{5}\right)+\left(1-p_{2}\right)p_{6}\left(1-p_{5}\right)+\left(1-p_{3}\right)\left(1-p_{6}\right)p_{5}+\left(1-p_{4}\right)p_{6}p_{5}}$$

According to the above relationship, we can calculate the likelihood of the sample using binomial distribution and execute the MCMC algorithm as follows:

#### MCMC algorithm

*X* is an *n* × 8 matrix including the numbers of variants of SNP1 and SNP2 in case and control in each study (*n* is the number of studies). *P* is an *n* × 3 matrix describing *p*_1_, *p*_5_ and *p*_6_ in each included study, and OR is a 1 × 3 vector containing OR_y,SNP1_, OR_y,SNP2_ and OR_interaction_. *X* is a known matrix, and *P* and OR are unknown matrices. *P* and OR can be expressed as follows:

$$P=\left[\begin{array}{ccc} p_{1,1} & p_{5,1} & p_{6,1}\\ p_{1,2} & p_{5,2} & p_{6,2}\\ p_{1,3} & p_{5,3} & p_{6,3}\\ \ldots & \ldots & \ldots \\ p_{1,n} & p_{5,n} & p_{6,n} \end{array}\right]$$

$$\text{OR}=\left[{\text{OR}}_{\;y,\text{SNP}1}\;{\text{OR}}_{\;y,\text{SNP}2}\;{\text{OR}}_{\;\text{interaction}}\right]$$

We can use the approach outlined in the following iteration process to construct a Markov chain stationary distribution Pr(*P*, OR| *X*) as follows:

#### Iteration process

Starting with initial values OR^(0)^ for OR (OR^(0)^ = [1 1 1]), we iterate the following steps for *m* = 1, 2, …

Step 1: Sample *P*^(m)^ from Pr(*P*^(m)^ |*X*, OR^(m−1)^)
Step 2: Sample OR^(m)^ from Pr(OR^(m)^ |*X*, *P*^(m)^)

In simple terms, *Step 1* is to assume that OR_y,SNP1_, OR_y,SNP2_ and OR_interaction_ are known parameters and to estimate *p*_1_, *p*_5_ and *p*_6_ in each included study using the Metropolis–Hastings algorithm. This algorithm will find the *p*_1_, *p*_5_ and *p*_6_ that maximise the likelihood of a given sample. Finally in this step, we can obtain the *p*_1_, *p*_5_ and *p*_6_ of each included study. *Step 2* is to assume that *p*_1_, *p*_5_ and *p*_6_ are known parameters and to estimate OR_y,SNP1_, OR_y,SNP2_ and OR_interaction_. We assume that each cell of *P* or OR is described by a random walk in the logistic or logarithmic normal distribution, respectively. The above two steps are repeated until convergence of the log likelihood.

### Implementation in ‘etma’ package by R language

An R package, etma, is developed for carrying out the epistasis detection in meta-analysis [etma is available via the Comprehensive R Archive Network (CRAN) at https://cran.r-project.org/web/packages/etma/index.html]. The main function of etma package is ‘ETMA’, and ETMA use an *n* × 8 matrix including the numbers of variants of SNP1 and SNP2 in case and control in each study (*n* is the number of studies) to analyse gene-gene interaction. Thus, the inputs of ETMA function include: (1) the number of wild type of SNP1 in case group, (2) the number of mutation type of SNP1 in case group, (3) the number of wild type of SNP1 in control group, (4) the number of mutation type of SNP1 in control group, (5) the number of wild type of SNP2 in case group, (6) the number of mutation type of SNP2 in case group, (7) the number of wild type of SNP2 in control group, and (8) the number of mutation type of SNP1 in control group.

Because ETMA is based on MCMC and a 2-steps iteration process (details are shown in 2.1 Derivations and description of ETMA). The main options of ETMA function include: (1) the maximum number of iterations (default is 20), (2) the length of chain to obtain the study-level parameters in step 1 (default is 20,000), (3) the length of chain to obtain the global-level parameters in step 2 (default is 200,000), and (4) the start seed of this algorithm (default is a random seed). Moreover, user also can choose whether want to export MCMC plots in each iterations.

**Fig 2:**
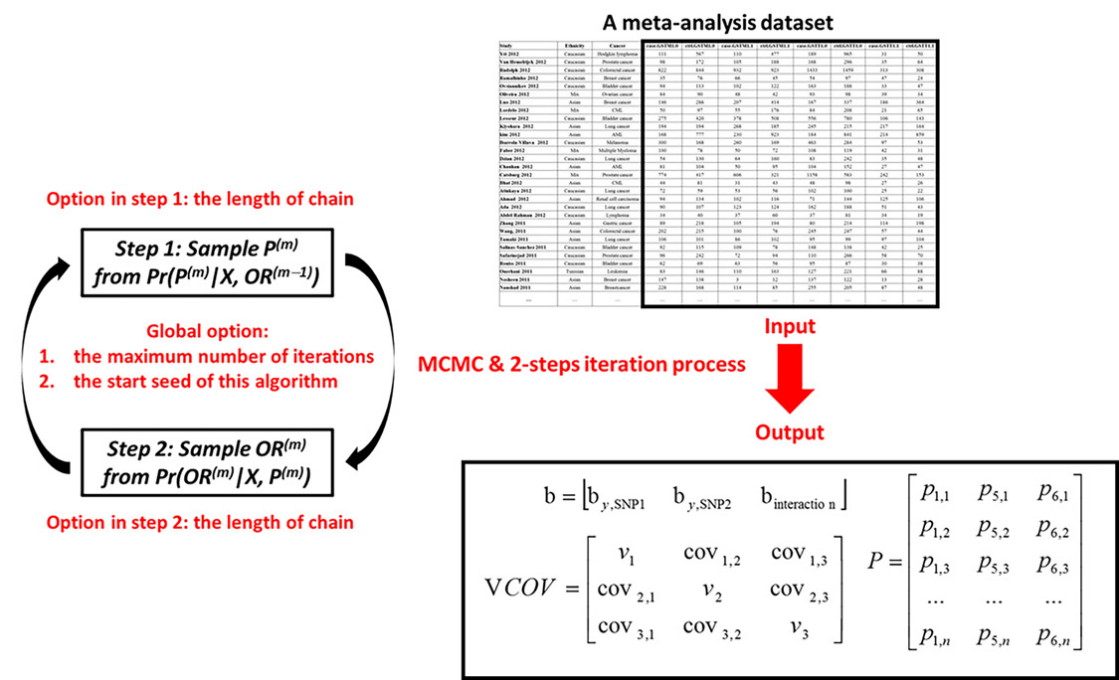
A typical analysis pipeline of ETMA function in 'etma' package. This figure summarized the pipeline of ETMA function. The main input is a meta-analysis dataset, which including the number of wild/mutation type of SNP1/SNP2 in case/control group. The main options include the length of chains in step 1/2, the maximum number of iterations, and the start seed. Main outputs include three matrixes. Matrix b includes the beta values (logarithmic ORs) of each SNP and interaction term, and VCOV is the variance covariance matrix of beta value. *P* is an n by 3 matrix describing three study-specific parameters (p1 = Disease risk in subjects with wild-type alleles of SNP1 and SNP2; p5 = Mutation frequency of SNP1; p6 = Mutation frequency of SNP2)

The main outputs include: (1) the beta values (logarithmic ORs) of each SNP and interaction term, (2) the variance covariance matrix of beta value, and (3) the p matrix in iterations process. According these outputs, we can calculate ORs, their confidence intervals, and p values. Fig 2 summarized the pipeline of ETMA function. Finally, a tutorial on epistasis detection using ETMA via ‘etma’ package is shown in S2 Text.

### Simulations

In this subsection, we simulated a meta-analysis of genetic association studies. In summary, we wanted to generate a data including population with different baseline disease risk and minor allele frequency. Moreover, ETMA is a method for analysing the meta-analysis of candidate genetic association studies, so we just need to generate 2 unlinkage SNPs (because the limit of summary data) and disease status. Follow above concept, we generated 20 large populations in each simulation, with three population-specific parameters: (1) the disease risk in subjects with wild-type alleles of SNP1 and SNP2 (*p*_baseline_), (2) the minor allele frequency of SNP1 (*MAF*_1_) and (3) the minor allele frequency of SNP2 (*MAF*_2_). We defined a series of *p*_baseline_ in our simulations, summarised in Table 1. The *MAF*_1_ and *MAF*_2_ were generated by the Balding–Nichols model [20]. We set the mean mutation frequency ($\bar{\pi }$) at 50% and fixed *F*_st_ at 0.1 in all simulations, and SNP1/SNP2 are independence and follow Hardy-Weinberg equilibrium. The minor allele frequency (*π*_i_) in each population was randomly generated from a beta distribution ($\alpha =\;\bar{\pi }\left(1-F_{\text{st}}\right)/F_{\text{st}}$; $\beta =\;\left(1-\bar{\pi }\right)\left(1-F_{\text{st}}\right)/F_{\text{st}}$). We defined three parameters descripting the effects of SNP1, SNP2 and their integration as OR_y,SNP1_, OR_y,SNP2_ and OR_interaction_, respectively, and the disease prevalence of individuals with different genotype of SNP1/SNP2 were following logistic regression. The values of OR_y,SNP1_, OR_y,SNP2_ and OR_interaction_ are summarised in Table 1. After we obtained *p*_baseline_, *MAF*_1_, *MAF*_2_, OR_y,SNP1_, OR_y,SNP2_ and OR_interaction_, the proportion of individual with different type of disease/SNP1/SNP2 could be calculated by Table 2. To use the information of Table 2, we randomly sampled a case–control study with a sample size randomly generated from a uniform (300, 1000) distribution. The proportion of cases was set to 50%.

**Table 1:** Summary of simulation conditions.

*p*_baseline_	OR_*y*,SNP1_	OR_*y*,SNP2_	OR_interaction_
~Uniform (0.001, 0.002)	1.0	1.0	1.0
~Uniform (0.01, 0.02)	1.2	1.2	1.2
~Uniform (0.1, 0.2)			1.5
			2.0

*p*_baseline_: the disease risk in subjects with major homozygous genotype of SNP1 and SNP2 in each simulated population.

OR_y,SNP1_: the main effect of SNP1.

OR_y,SNP2_: the main effect of SNP2.

OR_interaction_: gene–gene interaction effect between SNP1 and SNP2.

**Table 2:** The proportion of individual with different status of disease/SNP1/SNP2 could be calculated by *p*_baseline_, *MAF*_1_, *MAF*_2_, OR_y,SNP1_, OR_y,SNP2_ and OR_interaction_.

SNP1	SNP2	Disease	Proportion in total population
Major homozygous	Major homozygous	Control	(1-*MAF*_1_)^2^(1-*MAF*_2_)^2^(1-*q*_1_)
Major homozygous	Major homozygous	Case	(1-*MAF*_1_)^2^(1-*MAF*_2_)^2^*q*_1_
Major homozygous	Heterogeneous	Control	2(1-*MAF*_1_)^2^(1-*MAF*_2_)*p*_6_(1-*q*_2_)
Major homozygous	Heterogeneous	Case	2(1-*MAF*_1_)^2^(1-*MAF*_2_)*p*_6_*q*_2_
Major homozygous	Minor homozygous	Control	(1-*MAF*_1_)^2^(1-*MAF*_2_)^2^(1-*q*_3_)
Major homozygous	Minor homozygous	Case	(1-*MAF*_1_)^2^(1-*MAF*_2_)^2^*q*_3_
Heterogeneous	Major homozygous	Control	2*MAF*_1_(1-*MAF*_1_)(1-*MAF*_2_)^2^(1-*q*_4_)
Heterogeneous	Major homozygous	Case	2*MAF*_1_(1-*MAF*_1_)(1-*MAF*_2_)^2^*q*_4_
Heterogeneous	Heterogeneous	Control	4*MAF*_1_(1-*MAF*_1_)(1-*MAF*_2_)*p*_6_(1-*q*_5_)
Heterogeneous	Heterogeneous	Case	4*MAF*_1_(1-*MAF*_1_)(1-*MAF*_2_)*p*_6_*q*_5_
Heterogeneous	Minor homozygous	Control	2*MAF*_1_(1-*MAF*_1_)(1-*MAF*_2_)^2^(1-*q*_6_)
Heterogeneous	Minor homozygous	Case	2*MAF*_1_(1-*MAF*_1_)(1-*MAF*_2_)^2^*q*_6_
Minor homozygous	Major homozygous	Control	*MAF*_1_^2^(1-*MAF*_2_)^2^(1-*q*_7_)
Minor homozygous	Major homozygous	Case	*MAF*_1_^2^(1-*MAF*_2_)^2^*q*_7_
Minor homozygous	Heterogeneous	Control	2*MAF*_1_^2^(1-*MAF*_2_)*p*_6_(1-*q*_8_)
Minor homozygous	Heterogeneous	Case	2*MAF*_1_^2^(1-*MAF*_2_)*p*_6_*q*_8_
Minor homozygous	Minor homozygous	Control	*MAF*_1_^2^(1-*MAF*_2_)^2^(1-*q*_9_)
Minor homozygous	Minor homozygous	Case	*MAF*_1_^2^(1-*MAF*_2_)^2^*q*_9_

*p*_baseline_: the disease risk in subjects with major homozygous genotype of SNP1 and SNP2 in each simulated population; *MAF*_1_: the minor allele frequency of SNP1; *MAF*_2_: the minor allele frequency of SNP2; OR_y,SNP1_: the main effect of SNP1; OR_y,SNP2_: the main effect of SNP2; OR_interaction_: gene–gene interaction effect between SNP1 and SNP2.

*q*_1_ to *q*_9_: the disease prevalence of individuals with different genotype.

*q*_1_ = *p*_*baseline*_ = (1 + exp(−ln(*p*_*baseline*_ /(1 − *p*_*baseline*_))))^−1^ $q_{2}={\left(1+\text{exp}\left(-\text{ln}\left(p_{baseline}OR_{y,SNP_{2}}/\left(1-p_{baseline}\right)\right)\right)\right)}^{-1}$ $q_{3}={\left(1+\text{exp}\left(-\text{ln}\left(p_{baseline}OR_{y,SNP_{2}}{}^{2}/\left(1-p_{baseline}\right)\right)\right)\right)}^{-1}$ $q_{4}={\left(1+\text{exp}\left(-\text{ln}\left(p_{baseline}OR_{y,SNP_{1}}/\left(1-p_{baseline}\right)\right)\right)\right)}^{-1}$ $q_{5}={\left(1+\text{exp}\left(-\text{ln}\left(p_{baseline}OR_{y,SNP_{1}}OR_{y,SNP_{2}}/\left(1-p_{baseline}\right)\right)\right)\right)}^{-1}$ $q_{6}={\left(1+\text{exp}\left(-\text{ln}\left(p_{baseline}OR_{y,SNP_{1}}OR_{y,SNP_{2}}{}^{2}/\left(1-p_{baseline}\right)\right)\right)\right)}^{-1}$ $q_{7}={\left(1+\text{exp}\left(-\text{ln}\left(p_{baseline}OR_{y,SNP_{1}}{}^{2}/\left(1-p_{baseline}\right)\right)\right)\right)}^{-1}$ $q_{8}={\left(1+\text{exp}\left(-\text{ln}\left(p_{baseline}OR_{y,SNP_{1}}{}^{2}OR_{y,SNP_{2}}/\left(1-p_{baseline}\right)\right)\right)\right)}^{-1}$ $q_{9}={\left(1+\text{exp}\left(-\text{ln}\left(p_{baseline}OR_{y,SNP_{1}}{}^{2}OR_{y,SNP_{2}}{}^{2}/\left(1-p_{baseline}\right)\right)\right)\right)}^{-1}$

In the subsequent analysis, we compared three methods: ETMA, individual data analysis and conventional meta-analysis. The detailed calculation method of ETMA is described in section ‘Derivations and description of ETMA’, and this program used the summary data from each study. Individual data analysis is considered the gold standard for investigating the moderator effect [16,18], and we used a hierarchical generalised linear model based on the lme4 R package [21] with pooled data to estimate the interaction effect. Conventional meta-analysis was calculated based on a previous study [16]. Owing to the inconsistent estimates of interaction effects (refer to Fig 1), we used only the analysis fitting SNP1 as the independent variable and SNP2 as the moderator. Data under each condition were generated from 1,000 simulations.

### Application to real data

ETMA is a method for analysing the meta-analysis of candidate genetic association studies. Because the limit of multi-loci analysis technology, previous meta-analysis often focus on the association between a specific disease and a SNP but not on the epistasis. Thus, the existing meta-analysis including more than 1 SNP are rare. Moreover, only few papers completely provided their data, so such data is difficult to obtain. According to above reasons, we only can find 3 independent paper providing sufficient information for ETMA. It does not represent the practicability of ETMA is bad, but represent we need more meta-analysis investigating the epistasis.

#### Glutathione S-transferase (GST) family and cancer

The GST family detoxifies oxidative stress products, environmental toxins and carcinogens [22,23]. GST mu 1 (GSTM1) and GST theta 1 (GSTT1) are two critical GST family genes located in human chromosome regions 1p13.3 and 22q11.23, respectively. Generally, the variants in GSTM1 and GSTT1 are summarised as two types: (1) functional type and (2) null type [24–26]. Because of lack of detoxification mechanism, investigation of the associations between GSTM1/GSTT1 null type and cancer is popular. We used the data from a meta-analysis of approximately 500 studies investigating the association between GSTM1/GSTT1 and cancer [27] and selected the studies describing the genotypes of both GSTM1 and GSTT1. This filter left 360 studies (375 populations) in our real data analysis (the detailed data are shown in S1 Table).

#### Polycyclic aromatic hydrocarbons (PAHs) metabolism pathway and oral cancer

PAHs are strong carcinogens [28] found in coal tar, automobile exhaust fumes, charbroiled food and cigarette smoke. Cytochrome P450 1A1 (CYP1A1), located on chromosome 15, had been confirmed to be a component of the PAH metabolism pathway [29]. This pathway also involves the GST family. We used the data from a meta-analysis of approximately 50 studies investigating the association between CYP1A1/GSTM1 and oral cancer [30] and selected the studies describing the genotypes of both GSTM1 and CYP1A1 rs4646903. This filter left 13 studies in our real data analysis (the detailed data are shown in S2 Table).

#### Renin–angiotensin system (RAS) and chronic kidney disease

The RAS is a system-balancing electrolyte that regulates blood pressure, and a dysfunction of RAS increases the risk of kidney failure [31–33]. Angiotensinogen (AGT) is the initial protein in the RAS and is converted to angiotensin II, a terminal active product in the RAS [34]. This conversion is through renin and angiotensin-converting enzyme (ACE) [34]. We used the data from our earlier meta-analysis of approximately 100 studies investigating the association between ACE insertion/deletion (I/D) and chronic kidney disease [35] and selected the studies including AGT M235T information. We added four related articles published in 2014 [36–39]. There were then 34 studies in our real data analysis (the detailed data are shown in S3 Table).

## Results

### Simulation analysis

Table 3 shows the type I errors yielded by individual data analysis, ETMA and conventional meta-regression under each simulation condition. The type I errors of ETMA are between 0.033 and 0.052. In comparison with 0.05, ETMA was more conservative. The range of type I errors in individual data analysis and conventional meta-regression is 0.039–0.059 and 0.047–0.059, respectively. Thus, we judged all methods to have acceptable type I error. However, the meta-regression may have slight bias when the baseline disease risk is set to 0.1–0.2. This bias may be due to violation of the rare-disease assumption. A previous study showed a slight bias at a baseline disease risk equal to 0.1 [16].

**Table 3:** Type I error of individual data analysis, ETMA and conventional meta-regression.

Simulation conditions			Individual data analysis	ETMA	Conventional meta-regression
*p*_baseline_	OR_*y*,SNP1_	OR_*y*,SNP2_			
~Uniform (0.001, 0.002)	1.0	1.0	0.047	0.037	0.050
~Uniform (0.001, 0.002)	1.2	1.0	0.039	0.039	0.054
~Uniform (0.001, 0.002)	1.2	1.2	0.039	**0.034**	0.052
~Uniform (0.01, 0.02)	1.0	1.0	0.047	0.037	0.050
~Uniform (0.01, 0.02)	1.2	1.0	0.059	**0.033**	0.048
~Uniform (0.01, 0.02)	1.2	1.2	0.047	**0.034**	0.047
~Uniform (0.1, 0.2)	1.0	1.0	0.047	0.037	0.050
~Uniform (0.1, 0.2)	1.2	1.0	0.055	0.052	0.059
~Uniform (0.1, 0.2)	1.2	1.2	0.043	**0.033**	0.047

*p*_baseline_: the disease risk in subjects with major homozygous genotype of SNP1 and SNP2 in each simulated population.OR_y,SNP1_: the main effect of SNP1.

OR_y,SNP2_: the main effect of SNP2.

The bold value denotes a significant difference compared with 0.05 (the 95% confidence interval of type I error is between 0.036 and 0.064). Each data point was based on 1,000 simulations.

**Fig 3:**
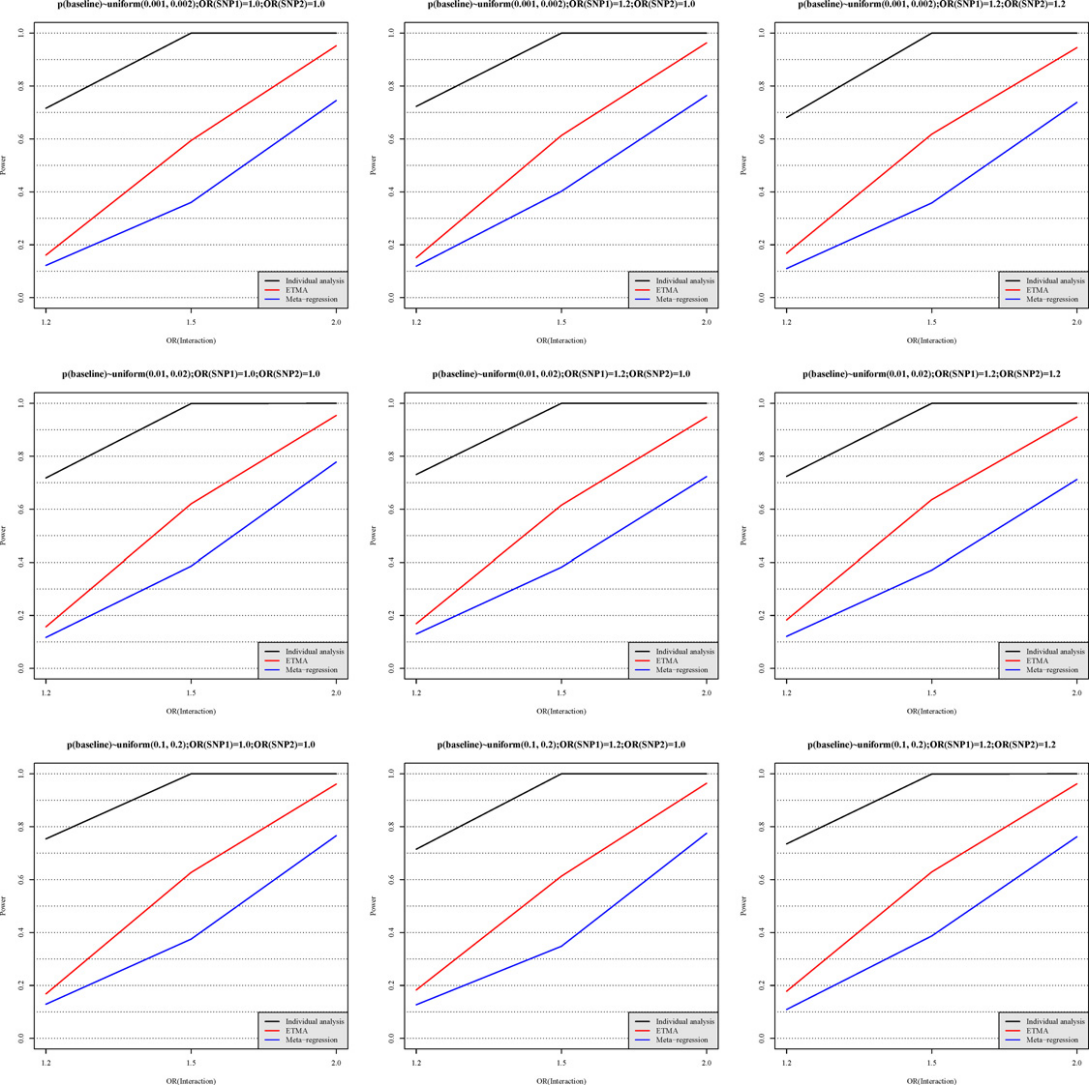
The statistical power of individual data analysis, ETMA and conventional meta-regression. The *x*-axis describes three levels of interaction effect (OR_interaction_ = 1.2, 1.5 or 2.0), and the *y*-axis indicates the statistical power provided by individual data analysis (black), ETMA (red) and conventional meta-regression (blue), respectively. The details of these methods are described in the Method. The different subplots present comparisons using different simulation parameters, and the titles of these subplots show their detailed settings. Each data point was based on 1,000 simulations.

Fig 3 shows the power of individual data analysis, ETMA and conventional meta-regression under each simulation condition. Overall, the performances of these three methods were not affected by the simulation conditions (*p*_1_, OR_y,SNP1_ and OR_y,SNP2_). In the power analysis, individual data analysis showed higher power than ETMA, followed by conventional meta-regression. The power of conventional meta-regression was slightly smaller when OR_y,SNP1_ and OR_y,SNP2_ were not equal to 1.0. This result may be due to damage of nonlinear relationship [16]. However, the power curves of ETMA were similar under all simulation conditions.

ETMA gave the higher statistical power compared with conventional meta-regression, and it also solved the challenge of inconsistent estimates (see Fig 1). Although individual data analysis gave the highest statistical power in our results, and previous evidence shows that individual data analysis is the gold standard [16,18,40]. The summary statistics are widely available [8,41], and individual information is difficult to obtain [10,42]. Thus, the practicability of ETMA is better than individual data analysis. In our simulation, the power of ETMA was higher than that of conventional meta-regression, and we considered the reason of higher power in ETMA as below: The first step of calculation in conventional meta-regression is to calculate OR from exposure rate [16]. We considered this step to represent a loss of information compared with ETMA. Moreover, given that our study showed a non-linear relationship between OR and mutation frequency, the linear relationship-based meta-regression was expected to give lower power.

Besides lower statistical power, conventional meta-regression must also face the challenge of inconsistent estimates. Although we ignored the second direction analysis in simulation, researchers will still be confused in real meta-analysis because inconsistent results will lead to difficulties of interpretation. In short, ETMA not only integrates the inconsistent information but also is more sensitive.

### Real data analysis

We applied ETMA to summary statistics from previous meta-analysis [27,30,35] (detailed information is presented in Methods). Table 4 shows the summary results of real data analysis (the detailed calculation process using the etma package is shown in S2 Text). For all studies, the logarithmic OR of SNP1, SNP2 and their interaction in the MCMC plot shows that normal distribution after burn-in time was deleted (the MCMC plots of the data sets are shown in S1–S3 Figs, respectively). Moreover, the marginal density plots show good convergence at each iteration. These results show that ETMA remains robust in analysis of real data.

**Table 4:** The result of real data analysis using ETMA.

Real data set		OR (95% CI)	p value
*GSTs family and cancer*			
	GSTM1 (null type vs. functional type)	1.110 (1.080–1.141)	<0.0001
	GSTT1 (null type vs. functional type)	1.125 (1.073–1.180)	<0.0001
	GSTM1×GSTT1 (interaction term)	0.942 (0.862–1.029)	0.1814
*Metabolism pathway of PAH and oral cancer*			
	CYP1A1 (AC/CC vs. AA)	0.819 (0.592–1.133)	0.2008
	GSTM1 (null type vs. functional type)	0.981 (0.717–1.340)	0.8915
	CYP1A1×GSTM1 (interaction term)	2.220 (1.166–4.225)	0.0201
*RAS and chronic kidney disease*			
	ACE (D allele vs. I allele)	0.921 (0.809–1.049)	0.2073
	AGT (T allele vs. M allele)	0.995 (0.884–1.120)	0.9277
	ACE ×AGT (interaction term)	1.305 (1.048–1.624)	0.0188

The result of analysis of the GST family and cancer shows significant ORs of GSTM1 and GSTM2 on cancer [1.110 (95% CI: 1.080–1.141) and 1.125 (95% CI: 1.073–1.180), respectively]. However, the interaction term of GSTM1 and GSTT1 is not significant (p = 0.2525). Although these genes belong to the same family, we also considered this to be a reasonable result. The GST family has many overlapping functions, and GSTM2 can perform more functions in subjects with a GSTM1 null genotype [43]. Moreover, the GSTM1/GSTT1 null genotype has been reported to confer a slight increase in risk [OR: 1.33 (95% CI: 1.10–1.61)] of lung cancer in a small-scale meta-analysis [11]. The result of our analysis was similar [OR: 1.176 (95% CI: 1.142–1.211); data are shown in S2 Text].

The analysis of the metabolism pathway of PAHs and oral cancer shows a significant gene–gene interaction effect (OR: 2.220 (95% CI: 1.166–4.225), *p* = 0.0201), and the main effect of each SNP is not significant (p = 0.2008 and 0.8915 for CYP1A1 and GSTM1, respectively). CYP1A1 and GSTM1 are two important members in the PAH metabolism pathway [29], and PAHs are strong carcinogens [28]. Moreover, a pooled analysis of lung cancer also reported a strong gene–gene interaction between them [44].

The analysis of the RAS and chronic kidney disease also shows a significant gene–gene interaction (OR: 1.305 (95% CI: 1.048–1.624), p = 0.0188). This result indicates an interaction effect between AGT M235T (rs699) and ACE I/D (rs4340) on chronic kidney disease, but that neither alone increases the risk of chronic kidney disease, because its main effect is not significant (p = 0.2073 and 0.9277 in ACE I/D and AGT M235T, respectively). The detailed mechanisms and possible reasons are described in the Discussion. We judged these results to be consistent with expectations. The AGT M235T polymorphism has been confirmed to affect blood AGT concentration [45], and excess AGT leads to a high concentration of angiotensin I in blood [46]. Moreover, the DD genotype of ACE I/D showed higher gene expression and serum ACE levels than the ID genotype, followed by the II genotype [47,48]. Thus, subjects carrying the T allele in AGT M235T and the D allele in ACE I/D may have especially high angiotensin II, based on the RAS pathway [34], and increased risk of chronic kidney disease [49]. In short, we propose that results of our real data analysis are consistent with current evidence.

## Discussion

Because the technological limitation of multi-loci analysis, previous meta-analysis often focus on the association between a specific disease and a SNP but not on the epistasis. Thus, the existing meta-analysis including more than 1 SNP are rare. However, epistasis is important in genetic association study. Previous studies considered that ‘missing heritability’ is often attributed to the technical limitations of epistasis estimation [3–5]. The summary statistics are widely available [8,41], and individual information is difficult to obtain [10,42]. ETMA have solved this technological limitation, and researchers can analyse gene-gene interaction using summary data. In this paper, we re-analysed few previous meta-analysis data [27,30,35], and found significant gene-gene interaction in PAHs metabolism pathway/RAS on oral cancer/chronic kidney disease. These findings may explain a part of‘missing heritability’ in oral cancer/chronic kidney disease, and improve our biological knowledge. We believe the multi-locus meta-analysis will be more popular in the future because this technological breakthroughs.

ETMA may lack the ability to detect gene–environment interactions because of issues related to degrees of freedom. ETMA is based on four exposure rates (of the *x*_1_ mutation in the case group, of the *x*_1_ mutation in the control group, of the *x*_2_ mutation in the case group and of the *x*_2_ mutation in the control group) in each included study. Some studies matched the environmental factors to reduce the confounding bias, sacrificing 1 degree of freedom. Thus, fitting of gene–environment interactions using ETMA will constitute overfitting. However, although this defect causes a problem in ETMA, it solves the problem of inconsistent estimates in meta-regression analysis [16]. Owing to matching, the odds ratios of environment factors are unavailable, so that gene–environment interaction analysis using meta-regression will yield a result for only one direction. Thus, we suggest that researchers use conventional meta-regression to detect gene–environment interaction [16] and ETMA to detect gene–gene interaction.

In conclusion, ETMA has acceptable type I error rates under all simulation condition. Moreover, it not only successfully facilitates consistency of evidence but also increases power. Although our results also show that individual data analysis is the most powerful analysis, sufficient detailed information is difficult to obtain, so that the practical value of ETMA for meta-analysis is higher. Because ETMA assumes independence between two loci, analysis of loci on different chromosomes is a better option (at least on different genes). For gene–environment interactions, we suggest that the researcher use conventional meta-regression unless it is verified that the distribution of environmental factors has not been artificially changed (such as by matching). Finally, a package (etma, readers can download it form https://cran.r-project.org/web/packages/etma/index.html) was developed in the R language and may be extensively applied to detect epistasis in meta-analyses.

## Supporting Information

S1 Fig ETMA of GSTM1/GSTT1 and cancer. Page 1 shows the MCMC plot for the first iteration, page 2 the second and so on. The final page shows the final iteration result, and the analysis results are based on this chain value.
(PDF)

S2 Fig ETMA of CYP1A1/GSTM1 and oral cancer. Page 1 shows the MCMC plot for the first iteration, page 2 the second and so on. The final page shows the final iteration result, and the analysis results are based on this chain value.
(PDF)

S3 Fig The ETMA of ACE/AGT and chronic kidney disease. Page 1 shows the MCMC plot for the first iteration, page 2 the second and so on. The final page shows the final iteration result, and the analysis results are based on this chain value.
(PDF)

S1 Table Meta-analysis data of GSTM1/GSTT1 on cancer from Fang et al. [27]. Variable definitions:
*Study*: the first author and published year in each included study
*Ethnicity*: the ethnicity of included population.
*Country*: the country of study.
*Cancer*: the cancer type.
*case*.*GSTM1*.*0*: the number of functional GSTM1 carriers in cases (including heterozygous).
*ctrl*.*GSTM1*.*0*: the number of functional GSTM1 carriers in controls (including heterozygous).
*case*.*GSTM1*.*1*: the number of null/null GSTM1 carriers in cases (risk type).
*ctrl*.*GSTM1*.*1*: the number of null/null GSTM1 carriers in controls (risk type).
*case*.*GSTT1*.*0*: the number of functional GSTT1 carriers in cases (including heterozygous).
*ctrl*.*GSTT1*.*0*: the number of functional GSTT1 carriers in controls (including heterozygous).
*case*.*GSTT1*.*1*: the number of null/null GSTT1 carriers in cases (risk type).
*ctrl*.*GSTT1*.*1*: the number of null/null GSTT1 carriers in controls (risk type).
(XLSX)

S2 Table Meta-analysis data of CYP1A1/GSTM1 on oral cancer from Liu et al. [30]. Variable definitions:
*Author*: the first author in each included article.
*Year*: the year of publication.
*Country*: the country of study location.
*case*.*CYP1A1*.*0*: the number of subjects with AA genotype in rs4646903 in cases.
*case*.*CYP1A1*.*1*: the number of subjects with AC/CC genotype in rs4646903 in cases (risk type).
*ctrl*.*CYP1A1*.*0*: the number of subjects with AA genotype in rs4646903 in controls.
*ctrl*.*CYP1A1*.*1*: the number of subjects with AC/CC genotype in rs4646903 in controls (risk type).
*case*.*GSTM1*.*0*: the number of functional GSTM1 carriers in cases (including heterozygous).
*case*.*GSTM1*.*1*: the number of null/null GSTM1 carriers in cases (risk type).
*ctrl*.*GSTM1*.*0*: the number of functional GSTM1 carriers in controls (including heterozygous).
*ctrl*.*GSTM1*.*1*: the number of null/null GSTM1 carriers in controls (risk type).
(XLSX)

S3 Table Meta-analysis data of ACE/AGT and chronic kidney disease from Lin et al. [35]. Variable definitions:
*Author*: the first author in each included article.
*Year*: the year of publication.
*Race*: the race of the study population.
*Type*: the subtype of chronic kidney disease in each study.
*case*.*ACE*.*0*: the number I allele in rs4340 in cases.
*case*.*ACE*.*1*: the number D allele in rs4340 in cases (risk type).
*ctrl*.*ACE*.*0*: the number I allele in rs4340 in controls.
*ctrl*.*ACE*.*1*: the number D allele in rs4340 in controls (risk type).
*case*.*AGT*.*0*: the number M allele in rs699 in cases.
*case*.*AGT*.*1*: the number T allele in rs699 in cases (risk allele).
*ctrl*.*AGT*.*0*: the number M allele in rs699 in controls.
*ctrl*.*AGT*.*1*: the number T allele in rs699 in controls (risk allele).
(XLSX)

S1 Text Detailed derivations of the relationships between *e*_case,x1_, *e*_ctrl,x1_, *e*_case,x2_ and *e*_ctrl,x2_ and *p*_1_, *p*_2_, *p*_3_, *p*_4_, *p*_5_ and *p*_6_. (DOCX)

S2 Text A tutorial on epistasis detection using ETMA. (DOCX)
