# Supplementary material for: Epistasis Test in Meta-Analysis: A Multi-Parameter Markov Chain Monte Carlo Model for Consistency of Evidence
Source: PLoS One. 2016 Apr 5;11(4):e0152891. doi: 10.1371/journal.pone.0152891 (PMC4821560; doi:10.1371/journal.pone.0152891)
Supplement: S1 Fig — Page 1 shows the MCMC plot for the first iteration, page 2 the second and so on. The final page shows the final iteration result, and the analysis results are based on this chain value. (PDF) [file pone.0152891.s001.pdf]

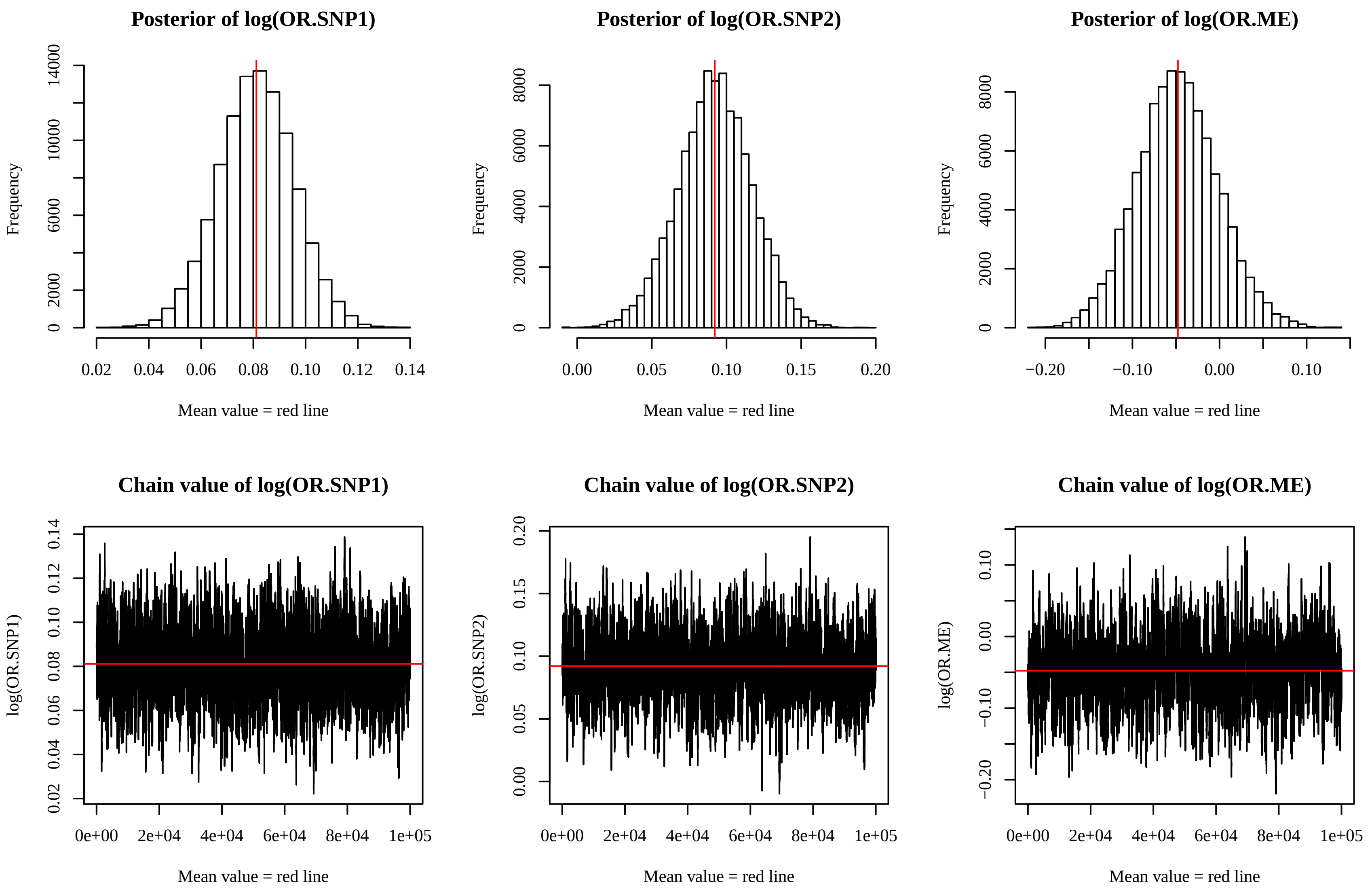

Posterior of log(OR.SNP1)
Posterior of log(OR.SNP2)
Posterior of log(OR.ME)
Frequency
Mean value = red line
Chain value of log(OR.SNP1)
Chain value of log(OR.SNP2)
Chain value of log(OR.ME)
log(OR.SNP1)
log(OR.SNP2)
log(OR.ME)
Mean value = red line

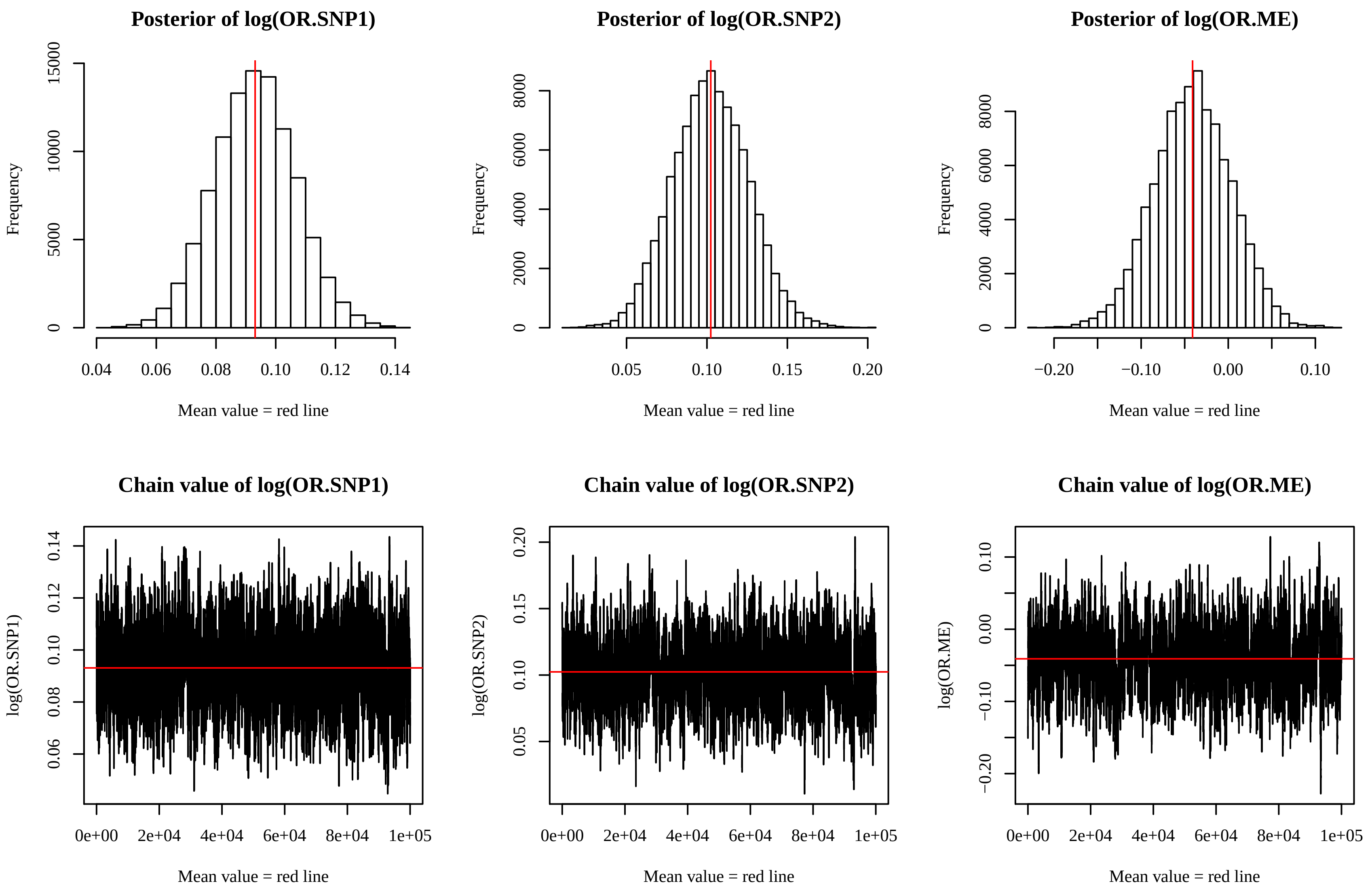
Posterior of log(OR.SNP1)
Frequency
0
5000
10000
15000
0.04
0.06
0.08
0.10
0.12
0.14
Mean value = red line
Posterior of log(OR.SNP2)
Frequency
0
2000
4000
6000
8000
0.05
0.10
0.15
0.20
Mean value = red line
Posterior of log(OR.ME)
Frequency
0
2000
4000
6000
8000
−0.20
−0.10
0.00
0.10
Mean value = red line
Chain value of log(OR.SNP1)
log(OR.SNP1)
0.06
0.08
0.10
0.12
0.14
0e+00
2e+04
4e+04
6e+04
8e+04
1e+05
Mean value = red line
Chain value of log(OR.SNP2)
log(OR.SNP2)
0.05
0.10
0.15
0.20
0e+00
2e+04
4e+04
6e+04
8e+04
1e+05
Mean value = red line
Chain value of log(OR.ME)
log(OR.ME)
−0.20
−0.10
0.00
0.10
0e+00
2e+04
4e+04
6e+04
8e+04
1e+05
Mean value = red line

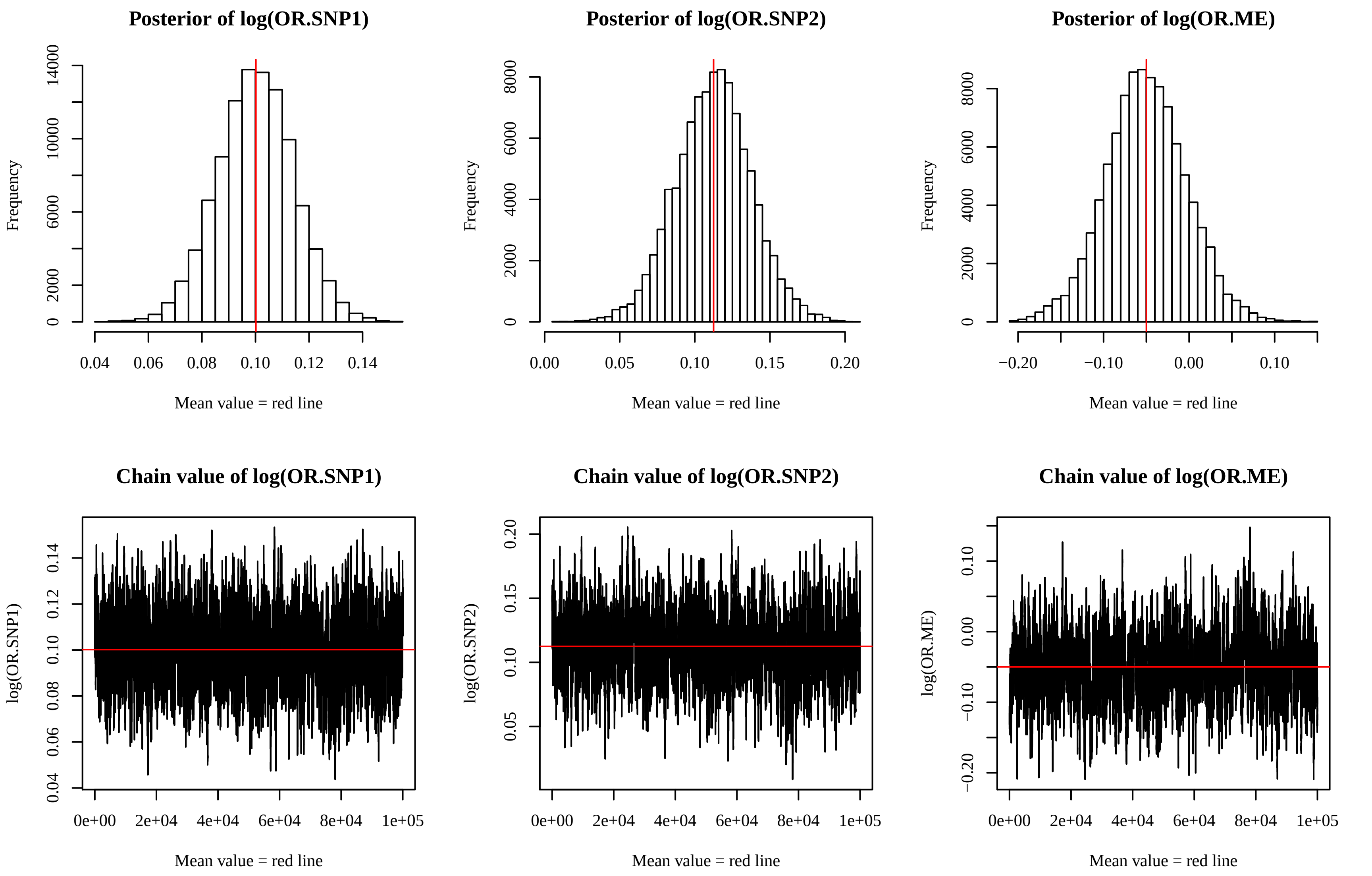

Posterior of log(OR.SNP1)
Posterior of log(OR.SNP2)
Posterior of log(OR.ME)
Frequency
0
2000
6000
10000
14000
0.04
0.06
0.08
0.10
0.12
0.14
0
2000
4000
6000
8000
0.00
0.05
0.10
0.15
0.20
−0.20
−0.10
0.00
0.10
Mean value = red line
Chain value of log(OR.SNP1)
Chain value of log(OR.SNP2)
Chain value of log(OR.ME)
log(OR.SNP1)
log(OR.SNP2)
log(OR.ME)
0e+00
2e+04
4e+04
6e+04
8e+04
1e+05

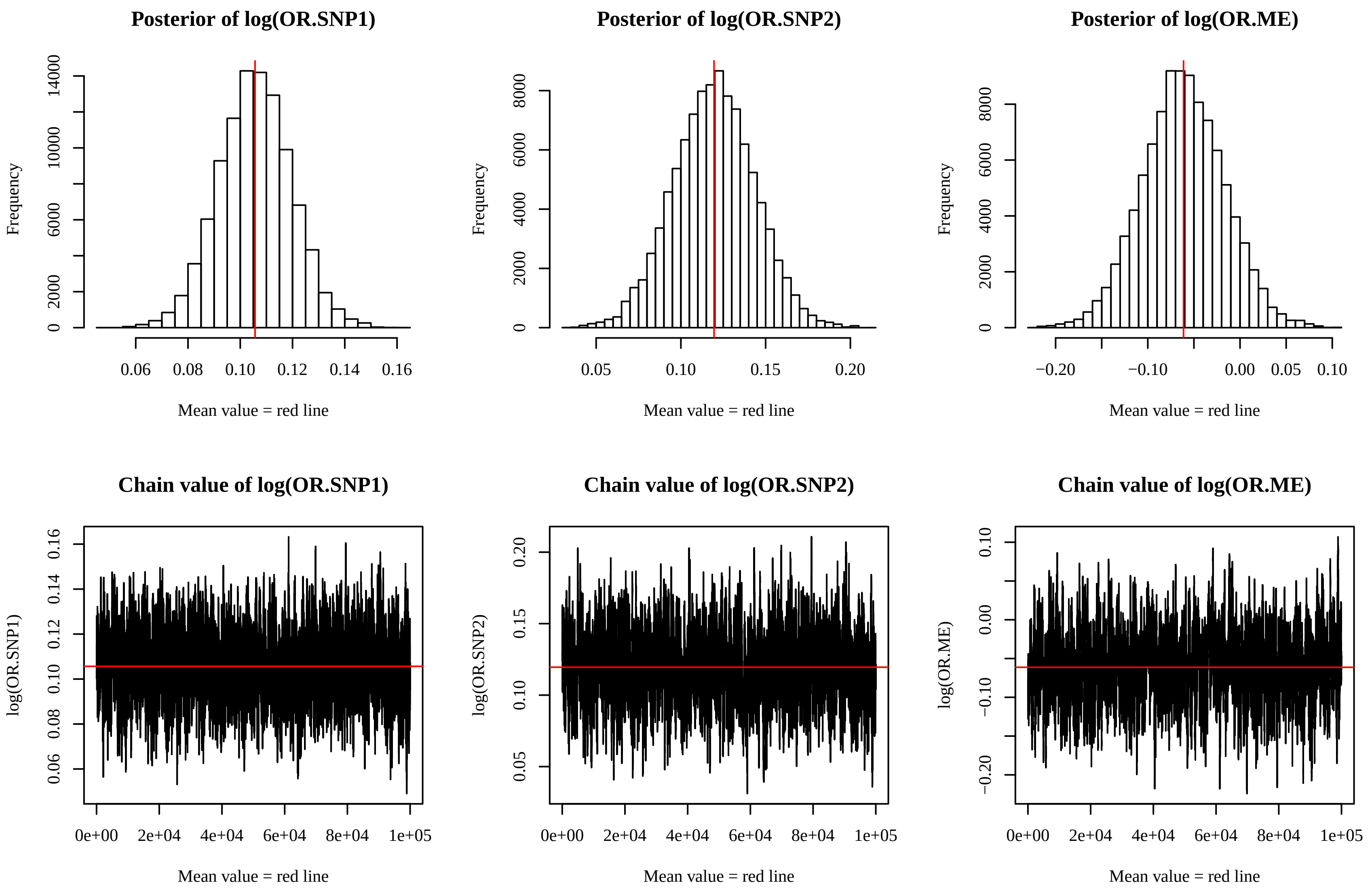
Posterior of log(OR.SNP1)
Frequency
0
2000
6000
10000
14000
0.06
0.08
0.10
0.12
0.14
0.16
Mean value = red line
Posterior of log(OR.SNP2)
Frequency
0
2000
4000
6000
8000
0.05
0.10
0.15
0.20
Mean value = red line
Posterior of log(OR.ME)
Frequency
0
2000
4000
6000
8000
−0.20
−0.10
0.00
0.05
0.10
Mean value = red line
Chain value of log(OR.SNP1)
log(OR.SNP1)
0.06
0.08
0.10
0.12
0.14
0.16
0e+00
2e+04
4e+04
6e+04
8e+04
1e+05
Mean value = red line
Chain value of log(OR.SNP2)
log(OR.SNP2)
0.05
0.10
0.15
0.20
0e+00
2e+04
4e+04
6e+04
8e+04
1e+05
Mean value = red line
Chain value of log(OR.ME)
log(OR.ME)
−0.20
−0.10
0.00
0.10
0e+00
2e+04
4e+04
6e+04
8e+04
1e+05
Mean value = red line

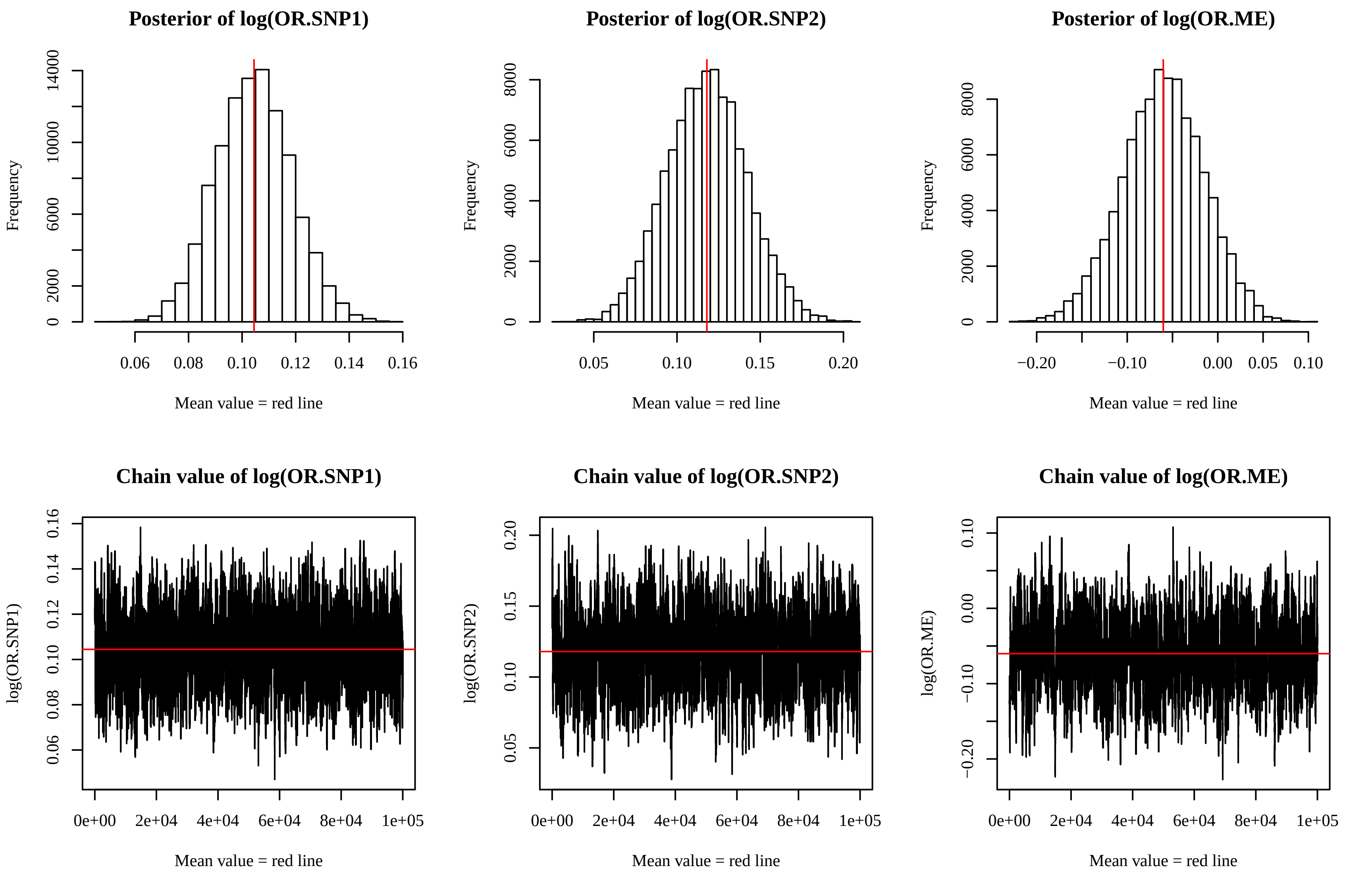
Posterior of log(OR.SNP1)
Frequency
0
2000
6000
10000
14000
0.06
0.08
0.10
0.12
0.14
0.16
Mean value = red line
Posterior of log(OR.SNP2)
Frequency
0
2000
4000
6000
8000
0.05
0.10
0.15
0.20
Mean value = red line
Posterior of log(OR.ME)
Frequency
0
2000
4000
6000
8000
−0.20
−0.10
0.00
0.05
0.10
Mean value = red line
Chain value of log(OR.SNP1)
log(OR.SNP1)
0.06
0.08
0.10
0.12
0.14
0.16
0e+00
2e+04
4e+04
6e+04
8e+04
1e+05
Mean value = red line
Chain value of log(OR.SNP2)
log(OR.SNP2)
0.05
0.10
0.15
0.20
0e+00
2e+04
4e+04
6e+04
8e+04
1e+05
Mean value = red line
Chain value of log(OR.ME)
log(OR.ME)
−0.20
−0.10
0.00
0.10
0e+00
2e+04
4e+04
6e+04
8e+04
1e+05
Mean value = red line
