# Supplementary material for: Epistasis Test in Meta-Analysis: A Multi-Parameter Markov Chain Monte Carlo Model for Consistency of Evidence
Source: PLoS One. 2016 Apr 5;11(4):e0152891. doi: 10.1371/journal.pone.0152891 (PMC4821560; doi:10.1371/journal.pone.0152891)
Supplement: S1 Text — (DOCX) [file pone.0152891.s007.docx]

**S1 Text. Detailed derivations of relationships between *e*_case,_*_x_*_1_, *e*_ctrl,_*_x_*_1_, *e*_case,_*_x_*_2_ and *e*_ctrl,_*_x_*_2_ and *p*_1_, *p*_2_, *p*_3_, *p*_4_, *p*_5_ and *p*_6_.**

**1. Exposure rate of *x*_1_ mutation in case group (*e*_case,_*_x_*_1_):**

$$\begin{matrix} e_{\text{case},x1}=p\left(x_{1}=1|y=1\right) \\ =\frac{p\left(y=1|x_{1}=1\right)p\left(x_{1}=1\right)}{p\left(y=1\right)} \\ =\frac{\left[p\left(y=1|x_{1}=1\cap x_{2}=0\right)p\left(x_{2}=0\right)+p\left(y=1|x_{1}=1\cap x_{2}=1\right)p\left(x_{2}=1\right)\right]p\left(x_{1}=1\right)}{p\left(y=1\right)} \\ =\frac{\left[p_{3}\left(1-p_{6}\right)+p_{4}p_{6}\right]p_{5}}{p\left(y=1\right)} \end{matrix}$$

**2. Exposure rate of *x*_1_ mutation in control group (*e*_ctrl,_*_x_*_1_):**

$$\begin{matrix} e_{\text{ctrl},x1}=p\left(x_{1}=1|y=0\right) \\ =\frac{p\left(y=0|x_{1}=1\right)p\left(x_{1}=1\right)}{p\left(y=0\right)} \\ =\frac{\left[p\left(y=0|x_{1}=1\cap x_{2}=0\right)p\left(x_{2}=0\right)+p\left(y=0|x_{1}=1\cap x_{2}=1\right)p\left(x_{2}=1\right)\right]p\left(x_{1}=1\right)}{p\left(y=0\right)} \\ =\frac{\left[\left(1-p_{3}\right)\left(1-p_{6}\right)+\left(1-p_{4}\right)p_{6}\right]p_{5}}{p\left(y=0\right)} \end{matrix}$$

**3. Exposure rate of *x*_2_ mutation in case group (*e*_case,_*_x_*_2_):**

$$\begin{matrix} e_{\text{case},x2}=p\left(x_{2}=1|y=1\right)= \\ \frac{p\left(y=1|x_{2}=1\right)p\left(x_{2}=1\right)}{p\left(y=1\right)} \\ =\frac{\left[p\left(y=1|x_{1}=0\cap x_{2}=1\right)p\left(x_{1}=0\right)+p\left(y=1|x_{1}=1\cap x_{2}=1\right)p\left(x_{1}=1\right)\right]p\left(x_{2}=1\right)}{p\left(y=1\right)} \\ =\frac{\left[p_{2}\left(1-p_{5}\right)+p_{4}p_{5}\right]p_{6}}{p\left(y=1\right)} \end{matrix}$$

**4. Exposure rate of *x*_2_ mutation in control group (*e*_ctrl,_*_x_*_2_):**

$$\begin{matrix} e_{\text{ctrl},x2}=p\left(x_{2}=1|y=0\right)= \\ \frac{p\left(y=0|x_{2}=1\right)p\left(x_{2}=1\right)}{p\left(y=0\right)} \\ =\frac{\left[p\left(y=0|x_{1}=0\cap x_{2}=1\right)p\left(x_{1}=0\right)+p\left(y=0|x_{1}=1\cap x_{2}=1\right)p\left(x_{1}=1\right)\right]p\left(x_{2}=1\right)}{p\left(y=0\right)} \\ =\frac{\left[\left(1-p_{2}\right)\left(1-p_{5}\right)+\left(1-p_{4}\right)p_{5}\right]p_{6}}{p\left(y=0\right)} \end{matrix}$$

Where *p*(*y* = 1), *p*(*y* = 0) is the disease risk and probability of disease-free in whole population, respectively. They can be calculated as follows:

**Disease risk in whole population [*p*(*y* = 1)]:**

$$p\left(y=1\right)=p_{1}\left(1-p_{6}\right)\left(1-p_{5}\right)+p_{2}p_{6}\left(1-p_{5}\right)+p_{3}\left(1-p_{6}\right)p_{5}+p_{4}p_{6}p_{5}$$

**Probability of disease-free status in whole population [*p*(*y* = 0)]:**

$$p\left(y=0\right)=\left(1-p_{1}\right)\left(1-p_{6}\right)\left(1-p_{5}\right)+\left(1-p_{2}\right)p_{6}\left(1-p_{5}\right)+\left(1-p_{3}\right)\left(1-p_{6}\right)p_{5}+\left(1-p_{4}\right)p_{6}p_{5}$$
