# Supplementary material for: Epistasis Test in Meta-Analysis: A Multi-Parameter Markov Chain Monte Carlo Model for Consistency of Evidence
Source: PLoS One. 2016 Apr 5;11(4):e0152891. doi: 10.1371/journal.pone.0152891 (PMC4821560; doi:10.1371/journal.pone.0152891)
Supplement: S2 Text — (DOCX) [file pone.0152891.s008.docx]

**S2 Text. A tutorial of epistasis detection using ETMA.**

*R* is a free and open-source language and computing environment, and the user can download it from the *R* home page (<http://www.r-project.org/>). After download and installation, the user may open the main *R* window and enter the following text to install *etma* package (assuming an internet connection and appropriate access rights on the computer):

*install.packages("etma")*

After installation, the user will need to enter the following text to load the *etma* package:

*library(etma)*

This package has a main function for detecting epistasis using ETMA (*ETMA*). To see the detailed arguments, use the *help* command as follows:

*help(etma)*

The *etma* package contains three complete example data sets. They are the data described in our article (section ‘Application to real data’). Use the *data* command to load these data and the *print* command to view them as follows. To analyze the data, use *help(read.table)* to view the details.

**GSTs family and cancer:**

*data(data.GST)*

*print(data.GST)*

**PAH metabolism pathway and oral cancer**

*data(data.PAH)*

*print(data.PAH)*

**RAS and chronic kidney disease**

*data(data.RAS)*

*print(data.RAS)*

The detailed definition of variables is presented in our article, or the user can use the *help* command to view the explanation. Use the *ETMA* command to analyze gene–gene interaction using ETMA and save the results to *ggint1*, *ggint2* and *ggint3*.

**GSTs family and cancer (note: the computing time for this example is about 3 h)**

*ggint1=ETMA(case.GSTM1.0,case.GSTM1.1,ctrl.GSTM1.0,ctrl.GSTM1.1,*

*case.GSTT1.0,case.GSTT1.1,ctrl.GSTT1.0,ctrl.GSTT1.1,*

*data=data.GST,start.seed=1,show.detailed.plot=TRUE,show.final.plot=TRUE)*

**PAH metabolism pathway and oral cancer (note: the computing time for this example is about 15 min)**

*ggint2=ETMA(case.CYP1A1.0,case.CYP1A1.1,ctrl.CYP1A1.0,ctrl.CYP1A1.1,*

*case.GSTM1.0,case.GSTM1.1,ctrl.GSTM1.0,ctrl.GSTM1.1,*

*data=data.PAH,start.seed=1,show.detailed.plot=TRUE,show.final.plot=TRUE)*

**RAS and chronic kidney disease (note: the computing time for this example is about 15 min)**

*ggint3=ETMA(case.ACE.0,case.ACE.1,ctrl.ACE.0,ctrl.ACE.1,*

*case.AGT.0,case.AGT.1,ctrl.AGT.0,ctrl.AGT.1,*

*data=data.RAS,start.seed=1,show.detailed.plot=TRUE,show.final.plot=TRUE)*

After the analysis, use the *print* and *summary* commands to view the result of gene–gene interaction analysis. (The red texts are the commands in the R window, and the blue texts are the output data.)

**GST family and cancer**

*print(ggint1)*

Epistasis Test in Meta-Analysis (ETMA)

A MCMC algorithm for detecting gene-gene interaction in meta-analysis.

This analysis include 375 studies. (df = 372)

b se OR 95%ci.l 95%ci.u t value p value

SNP1 (mutation) 0.10446 0.01406 1.110 1.080 1.141 7.4310 <0.0001

SNP2 (mutation) 0.11796 0.02402 1.125 1.073 1.180 4.9106 <0.0001

Interaction -0.06020 0.04497 0.942 0.862 1.029 -1.3388 0.1814

*summary(ggint1)*

Epistasis Test in Meta-Analysis (ETMA)

A MCMC algorithm for detecting gene-gene interaction in meta-analysis.

This analysis include 375 studies. (df = 372)

b se OR 95%ci.l 95%ci.u t value p value

SNP1 (mutation) 0.10446 0.01406 1.110 1.080 1.141 7.4310 <0.0001

SNP2 (mutation) 0.11796 0.02402 1.125 1.073 1.180 4.9106 <0.0001

Interaction -0.06020 0.04497 0.942 0.862 1.029 -1.3388 0.1814

OR 95%ci.l 95%ci.u t value p value

SNP1 (wild type) and SNP2 (mutation) 1.125 1.073 1.180 4.9106 <0.0001

SNP1 (mutation) and SNP2 (wild type) 1.110 1.080 1.141 7.4310 <0.0001

SNP1 (mutation) and SNP2 (mutation) 1.176 1.142 1.211 10.8983 <0.0001

**PAH metabolism pathway and oral cancer**

*print(ggint2)*

Epistasis Test in Meta-Analysis (ETMA)

A MCMC algorithm for detecting gene-gene interaction in meta-analysis.

This analysis include 13 studies. (df = 10)

b se OR 95%ci.l 95%ci.u t value p value

SNP1 (mutation) -0.19967 0.14580 0.819 0.592 1.133 −1.3695 0.2008

SNP2 (mutation) -0.01963 0.14025 0.981 0.717 1.340 −0.1400 0.8915

Interaction 0.79747 0.28886 2.220 1.166 4.225 2.7608 0.0201

*summary(ggint2)*

Epistasis Test in Meta-Analysis (ETMA)

A MCMC algorithm for detecting gene-gene interaction in meta-analysis.

This analysis include 13 studies. (df = 10)

b se OR 95%ci.l 95%ci.u t value p value

SNP1 (mutation) -0.19967 0.14580 0.819 0.592 1.133 -1.3695 0.2008

SNP2 (mutation) -0.01963 0.14025 0.981 0.717 1.340 -0.1400 0.8915

Interaction 0.79747 0.28886 2.220 1.166 4.225 2.7608 0.0201

OR 95%ci.l 95%ci.u t value p value

SNP1 (wild type) & SNP2(mutation) 0.981 0.717 1.340 -0.1400 0.8915

SNP1 (mutation) & SNP2(wild type) 0.819 0.592 1.133 -1.3695 0.2008

SNP1 (mutation) & SNP2(mutation) 1.783 1.506 2.110 7.6478 <0.0001

**RAS and chronic kidney disease (note: the computing time for this example is about 10 min)**

*print(ggint3)*

Epistasis Test in Meta-Analysis (ETMA)

A MCMC algorithm for detecting gene-gene interaction in meta-analysis.

This analysis include 34 studies. (df = 31)

b se OR 95%ci.l 95%ci.u t value p value

SNP1 (mutation) -0.08189 0.06358 0.921 0.809 1.049 -1.2879 0.2073

SNP2 (mutation) -0.00530 0.05799 0.995 0.884 1.120 -0.0914 0.9277

Interaction 0.26613 0.10729 1.305 1.048 1.624 2.4803 0.0188

*summary(ggint3)*

Epistasis Test in Meta-Analysis (ETMA)

A MCMC algorithm for detecting gene-gene interaction in meta-analysis.

This analysis include 34 studies. (df = 31)

b se OR 95%ci.l 95%ci.u t value p value

SNP1 (mutation) -0.08189 0.06358 0.921 0.809 1.049 −1.2879 0.2073

SNP2 (mutation) -0.00530 0.05799 0.995 0.884 1.120 −0.0914 0.9277

Interaction 0.26613 0.10729 1.305 1.048 1.624 2.4803 0.0188

OR 95%ci.l 95%ci.u t value p value

SNP1 (wild type) and SNP2 (mutation) 0.995 0.884 1.120 −0.0914 0.9277

SNP1 (mutation) and SNP2 (wild type) 0.921 0.809 1.049 −1.2879 0.2073

SNP1 (mutation) and SNP2 (mutation) 1.196 1.123 1.274 5.7782 <0.0001
